# Deep eutectic solvents for solid pesticide dosage forms

**DOI:** 10.1038/s41598-020-77559-0

**Published:** 2020-11-26

**Authors:** Justin Phillips, Walter W. Focke, Elizabeth L. du Toit, James Wesley-Smith

**Affiliations:** 1 Department of Chemical Engineering, Institute of Applied Materials, University of Pretoria, Hatfield, Private Bag X20, Pretoria, 0028 South Africa; 2 Sefako Makgatho Health Sciences University, Molotlegi St, Ga-Rankuwa Zone 1, Ga-Rankuwa, 0208 South Africa

**Keywords:** Chemistry, Materials science

## Abstract

Deep eutectic solvents aid the formulation of solid pesticide dosage forms for water-insoluble actives. This was demonstrated by encapsulating Amitraz powder in a low-melting matrix based on the eutectic mixture of urea (32 wt%) and 1,3-dimethylurea. Dissolution in water of melt-cast discs, containing 20 wt% active, led to the rapid release of Amitraz in a finely dispersed form. The order of magnitude reduction in particle size, after dissolution, is ascribed to the solubilization of Amitraz in the hot deep eutectic solvent and its subsequent precipitation as a separate phase on crystallization of the matrix.

## Introduction

Controlled-release formulations for pesticide applications act as depot systems that continuously release the active ingredients into the environment over a specified period, usually from months to years^1,2^. However, some applications require fast-dissolving drug delivery^3^. Our interest is in fast-release of water-insoluble pesticides into aquatic environments. The focus is on matrix-based dosage forms such as tablets, granules or fibers that either disintegrate or dissolve to release a water-insoluble active. These types of dosage forms can be fabricated using processes such as lyophilization, spray drying, solvent casting, hot melt extrusion^2,4^, compression molding, wet granulation, compaction and electrospinning^3,5^. However, here a simple melt-casting procedure is described.

Deep eutectic solvents (DESs) are environmentally friendlier alternatives to ionic liquids. They comprise two or more components that are capable of self-association, often through hydrogen bond interactions^6^. DESs exhibit physio-chemical properties similar to those of ionic liquids, e.g. negligible vapor pressure, while being much less expensive^7^. The eutectic composition has a melting point lower than that of each parent component and DESs are generally liquid at temperatures lower than 100 °C^6,8^. Recently, Suriyanarayanan^8^ investigated a family of non-ionic deep eutectic liquids based upon mixtures of acetamide and solid derivatives of urea. However, Soviet scientists were the first to study mixtures of the environmentally benign and non-toxic substances urea and acetamide^8^. The eutectic corresponds approximately to 33 wt% urea/67 wt% acetamide and the melting point was indicated as 329 K. This communication reports on the utility of a similar system based on urea together with 1,3-dimethylurea. The potential of deep eutectic solvents for formulating solid dosage forms, suitable for water insoluble actives, is demonstrated with this system. Amitraz, which is a triazapentadiene acaricide, is an example of such an active ingredient. The non-toxic nature, high water solubility and relatively low cost of urea and 1,3-dimethylurea imply that Amitraz in the proposed solid dosage form may have potential application in plunge dip treatments of livestock.

## Results

### Differential scanning calorimetry (DSC)

**Figure 1:**
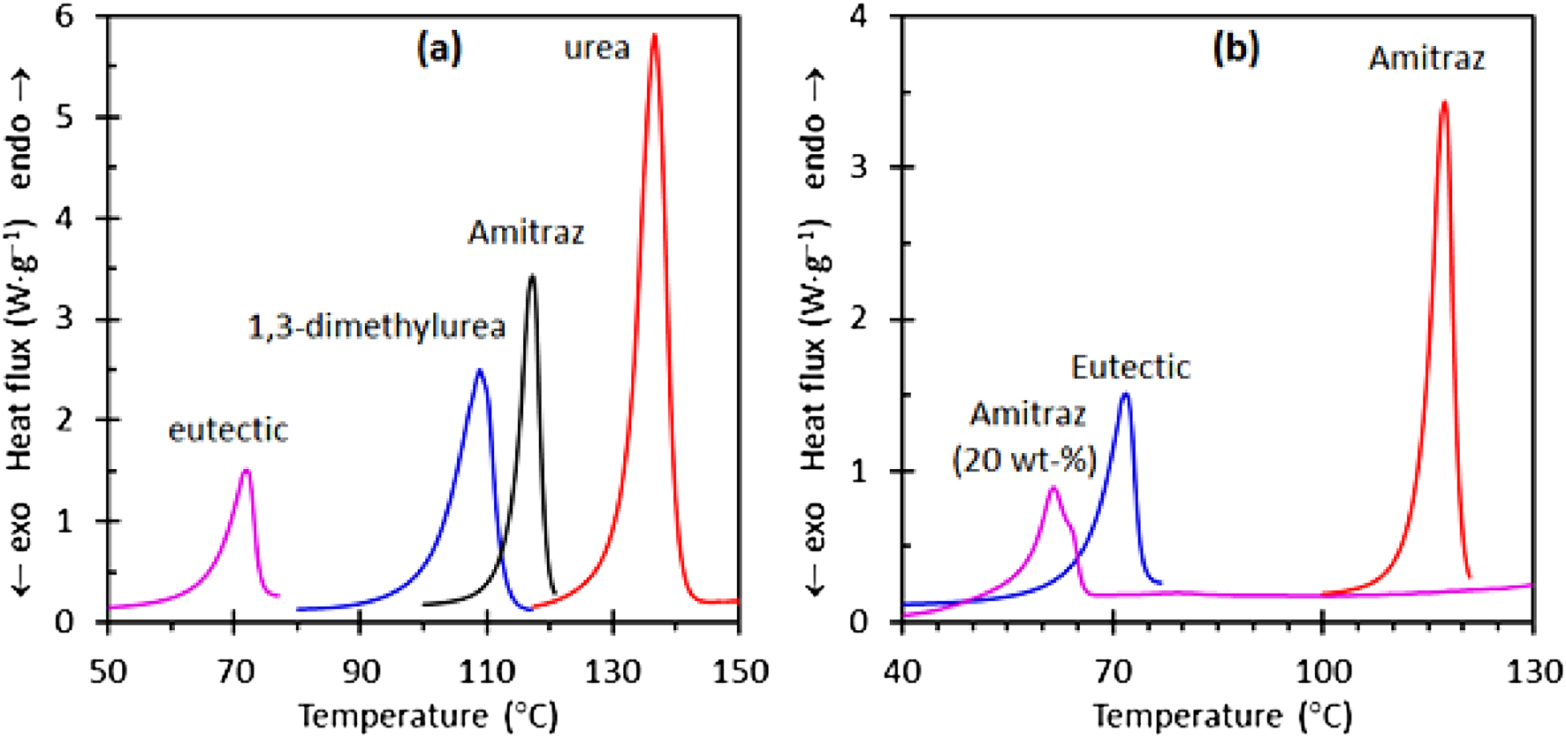
DSC melting endotherms obtained at a scan rate of 10 K min^−1^. (**a**) Results for urea, Amitraz, 1,3-dimethylurea and the eutectic composition. (**b**) Comparing the melting endotherm of the eutectic filled with 20 wt% Amitraz to the endotherms for the pure eutectic and the neat Amitraz.

Figure 1a reports the DSC melting endotherm results for the neat compounds and the eutectic composition. Figure 1b compares the melting endotherms of the 20 wt% Amitraz composition to those of the neat eutectic and the pure Amitraz. The melting onset temperatures for the urea and the 1,3-dimethylurea were found to be 131.5 °C and 101.5 °C respectively. The DSC indicated melting temperature for the eutectic was 60 °C.

### Urea: 1,3-dimethylurea phase diagram

**Figure 2:**
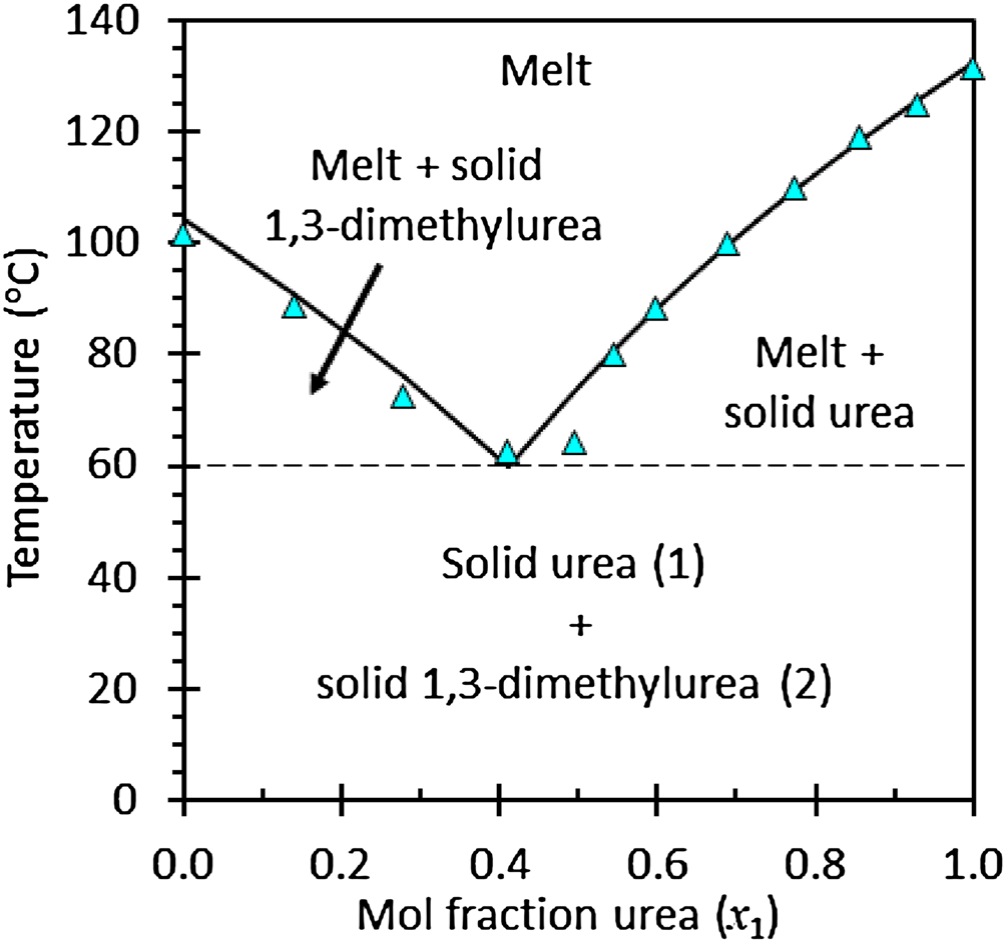
Liquid–solid phase diagram for the urea (1)—1,3-dimethylurea (2) system.

Figure 2 shows the phase diagram that was constructed on the basis of the recorded cooling curves and DSC results. The data were regressed using Margules-based activity coefficients. This yielded a eutectic composition estimate of 32 wt% urea (or 41 mol% urea). These results differ somewhat from the values reported by Suriyanarayanan^8^. They reported a eutectic temperature of 69 ± 3 °C at a composition of 30 wt% urea.

### Crystal morphology

**Figure 3:**
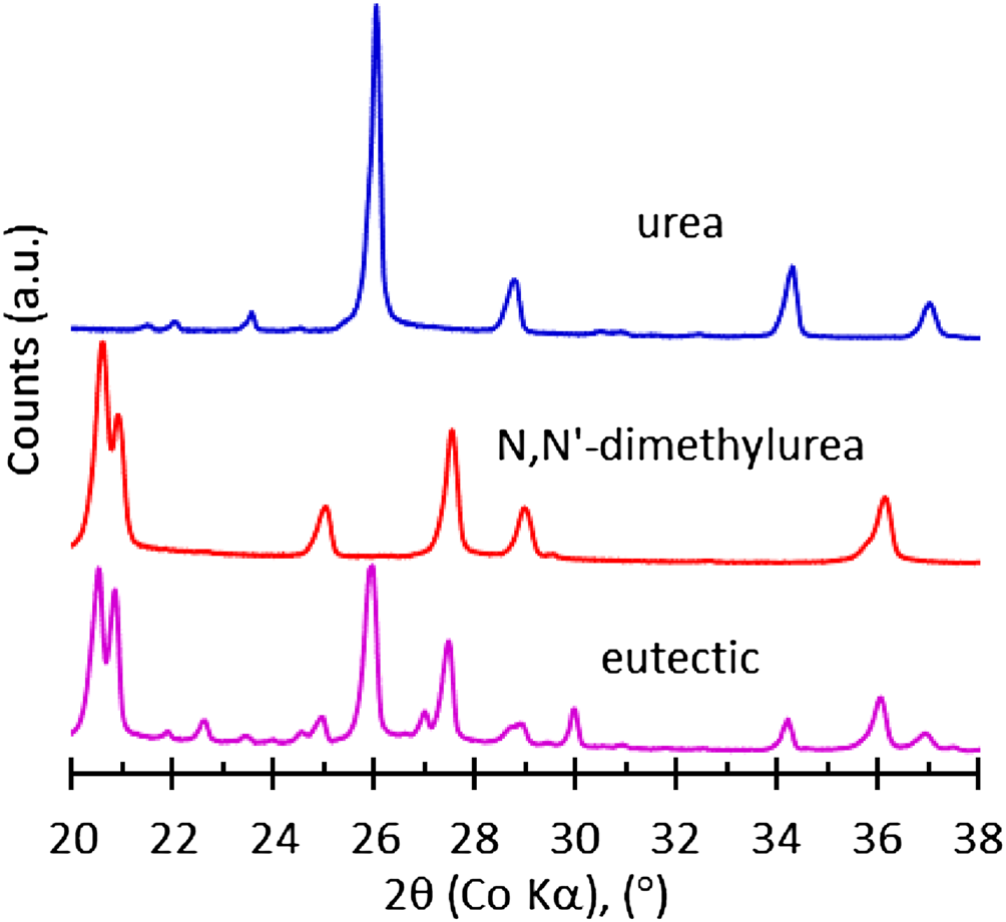
X-ray diffractograms of pure component crystals and eutectic mixture crystals.

Figure 3 displays the X-ray diffractograms obtained for the eutectic compound and the two parent compounds. The pure components display clear and distinct reflections, which are also present in the diffractogram obtained for the eutectic composition. In addition, the latter features three new low-intensity reflections at 2θ = 22.5°, 25° and 30°. This may indicate that a minor amount of co-crystallization of urea and 1,3-dimethylurea occurred, most likely at the grain boundaries where the growing pure component crystals impinged on one another.

### Dissolution in water

**Figure 4:**
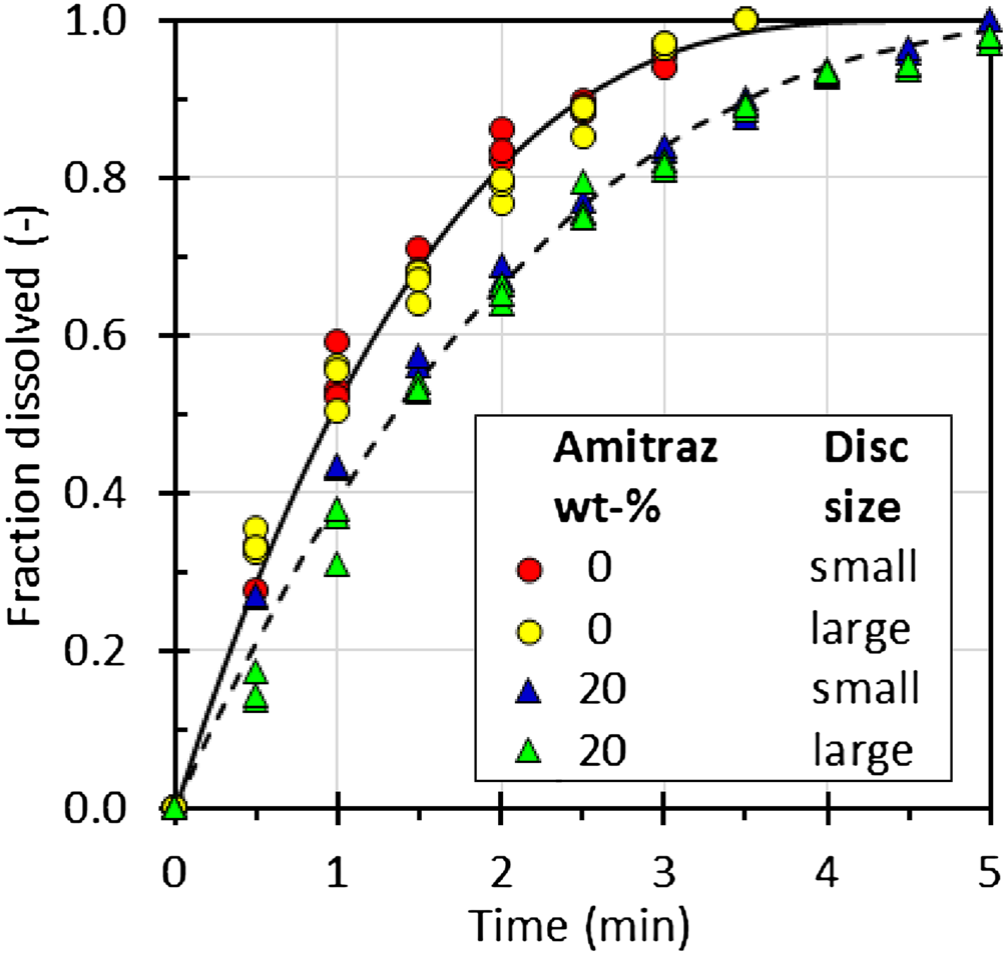
The effect of disc size on the dissolution time of the neat eutectic mixture and a formulation containing 20 wt% Amitraz. The lines show fits of the Hixson-Crowell cube root law (Eq. 1).

Figure 4 shows the dissolution results for both small and large discs of the neat eutectic as well as of the formulation that contained 20 wt% Amitraz. The data trends were consistent with the Hixson-Crowell cube root law^9^

1 $$ \left[ {M\left( t \right)/M_{o} } \right]^{1/3} = 1 - {t \mathord{\left/ {\vphantom {t \tau }} \right. \kern-\nulldelimiterspace} \tau } $$

where *M*_*o*_ is the initial mass; *M*(*t*) is the mass remaining after time *t*, and τ is a characteristic time constant indicative of the time required for complete dissolution. Surprisingly, the dissolution time τ was unaffected by the disc diameter but it was shorter when Amitraz was present (τ = 6.56 min; 95% confidence interval (6.38, 6.78)) compared to the neat eutectic system (τ = 4.65 min; 95% confidence interval (4.55, 4.75)). The disc thickness was the same in both cases and the aspect ratios were relatively large (11 and 20). Therefore, the diameter-independent dissolution time probably indicates that the large exposed circular surface controlled the dissolution rate with the much smaller surface area of the disc edges playing a minor role.

### Particle size distributions

**Figure 5:**
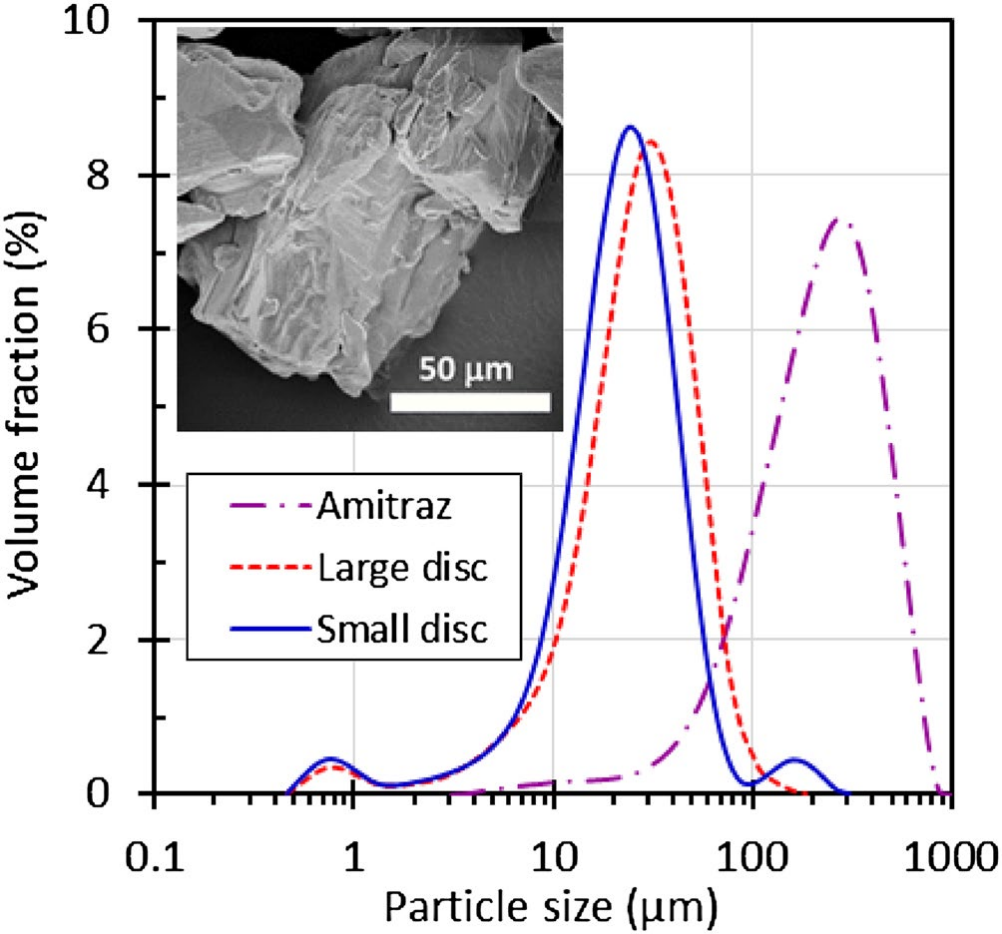
Particle size distributions for the neat Amitraz powder and dispersions obtained after dissolution of formulations containing 20 wt% Amitraz. The insert shows a SEM micrograph of an Amitraz powder particle.

Figure 5 compares the particle size distributions of the aqueous Amitraz dispersions with that of the original neat powder. The D_10_, D_50_ and D_90_ particle sizes are listed in Table 1. The key finding is an almost order-of-magnitude reduction in the Amitraz particle size as released from the solid dosage system. The D_50_ particle size of the neat Amitraz powder was 240 ± 10 µm. After dissolution of the solid dosage form, the D_50_ particle size of the Amitraz dispersion was less than 30 µm.

**Table 1:** Particle size values for the neat Amitraz powder and for dispersions obtained after dissolution of cast discs.

Sample	D_10_ (μm)	D_50_ (μm)	D_90_ (μm)
Amitraz (neat)	81 ± 4	240 ± 10	511 ± 28
Dispersion from small disc	9.3 ± 0.1	25 ± 1	50 ± 2
Dispersion from large disc	10.3 ± 0.3	30 ± 3	60 ± 12

## Discussion

There are two possible explanations for the observed results. The large particles indicated by the particle size distribution results could in fact have been agglomerates of much smaller constituent particles. It could then be, that the processing that happened during the preparation of the Amitraz-filled eutectic effected their dispersion as free individual particles. Solubilization of the Amitraz by the molten deep eutectic solvent presents a second alternative explanation. In this scenario, phase separation occurred during cooling of the homogeneous solution on or before solidification. This resulted in the formation of small Amitraz domains that solidified as separate, much smaller particles trapped inside the eutectic matrix. These were subsequently released during the dissolution process.

Both SEM results and DSC information favor the second interpretation. Figure 1b shows that the eutectic, filled with 20 wt% Amitraz, featured only one major endotherm. It is located at a slightly lower temperature range than the one observed for the urea—1,3-dimethylurea eutectic. This, and the absence of a second endotherm closer to the melting point of the Amitraz indicates that the active did indeed dissolve in the deep eutectic solvent. This led to a further lowering of the melting point of the eutectic. Finally, the SEM micrograph, shown as an insert in Fig. 5, shows that the neat Amitraz powder consisted of monolithic particles exceeding 100 μm in size. This means that, although the neat Amitraz particles might have been partially agglomerated and difficult to disperse, the actual particles released on dissolution must have derived from a recrystallization from the deep eutectic solvent.

## Conclusions

Nonionic deep eutectic solvents show potential as matrix materials useful for formulating rapid release dosage forms for water-insoluble pesticides. Advantages include low temperature (< 100 °C) processing and mixing of the ingredients; the possibility of direct casting of the required tablet shapes by pouring the molten mixture into the plastic packaging containers used for storage and transport; rapid release in water media via dissolution of the matrix; the automatic generation of a finely dispersed forms of the active through the process of melting the deep eutectic solvent, the dissolution of the active and its phase separation on cooling and solidification of the eutectic. This implies that fine grinding of the actives might not be necessary. The above features were exemplified using the acaricide Amitraz incorporated into the urea—1,3-dimethylurea eutectic.

## Materials and methods

### Materials selection

Sigma-Aldrich supplied urea and 1,3-dimethylurea with stated purities of 98% and 99% respectively. They were used as received, i.e. without further purification. The Animal Health Division of Bayer (Pty) LTD (South Africa) supplied finely milled technical grade Amitraz powder, with a purity of 98.5%.

### Differential scanning calorimetry (DSC)

The melting endotherms of the raw materials and the urea—1,3-dimethylurea eutectic, in neat form as well as filled with the acaricide, were determined by differential scanning calorimetry using a PerkinElmer Pyris 6 DSC. Samples weighing 12 ± 3 mg were sealed in aluminum pans with pinhole lids. The temperature was twice cycled from 20 °C to 140 °C and back. The heating rate was 10 K min^−1^ with nitrogen flowing at 20 mL min^−1^. Only the melting events recorded in the second heating cycle are reported here. The temperature calibration of the DSC instrument was checked using an indium standard.

### Cooling curves

Cooling curves were generated in order to identify the eutectic composition. The procedure was as follows: Predetermined quantities of urea and 1,3-dimethylurea were weighed into a test tube. The test tube was partially submerged in silicon oil contained in a beaker placed on a heater-stirrer. The contents were heated until the test tube contents were fully molten. The molten mixture was agitated with a glass rod to ensure complete homogenization of the contents. After the heater-stirrer was switched off, the change in temperature with time was tracked with a Hairuis Model SSN-61 temperature data logger. Care was taken that the thermocouple inserted into the test tube did not touch the glass walls. In most cases, sub-cooling occurred as indicated by a sudden rise in the temperature when freezing commenced. The peak temperature measured, following this event, was taken to correspond to the equilibrium crystallization temperature for the corresponding composition. Duplicate runs were conducted for each composition considered.

### X-ray diffraction

Samples of urea and 1,3-dimethylurea as well as the eutectic mixture were pulverized with a mortar and pestle. The powder samples were stored over silica gel absorbent in a desiccator for at least 12 h before analyses. Near randomly oriented samples were prepared by means of the PANalytical backloading system. X-ray powder diffraction data was obtained on a PANalytical X’Pert Pro diffractometer in the θ-θ configuration using an X’Celerator detector with variable divergence- and fixed receiving slits. Fe-filtered Co Kα radiation (λ = 1.789 Å) was used.

#### Preparation of solid dosage forms

The deep eutectic solvent was prepared by heating powder mixtures of urea and 1,3-dimethyl urea, corresponding to the eutectic composition, for approximately 1 h in an oven set at a temperature of 70 °C. The Amitraz solid dosage form was obtained by dissolving the acaricide in the hot solvent.

Test discs for the dissolution experiments were cast by pouring the prepared solutions into two different-sized polypropylene cups. These served as the molds that facilitated the casting of discs with different diameters (55 mm ϕ and 100 mm ϕ) but with identical thicknesses (5.0 mm). Predetermined quantities of the homogeneous liquids were poured into the molds and allowed to cool and solidify over a period of 1 h in a refrigerator set at a temperature of 2 °C. The castings were removed from the molds and stored in an airtight container. Casts were made using the neat eutectic composition as is, and also with the Amitraz pesticide powder mixed-in at a ratio of 1:4 by mass.

### Dissolution trials

The dissolution of the discs was tracked gravimetrically. Duplicate determinations were made according to the following procedure. Prior to each experiment, the discs were weighed using a Radwag PS 360/C/2 laboratory scale. They were then placed in a 2 L beaker filled with 1 L of deionized water, which was agitated with an overhead WiseStir HS-120A stirrer set to 200 rpm. The discs were temporarily removed and weighed at regular intervals.

### Particle size analysis

Particle size analysis was conducted on a Malvern Mastersizer 3000. The wet dispersion of the solid powders for particle size analysis was controlled with a Malvern LV Hydro. The ultrasound setting was 20% and the shear mixer was set at 3500 rpm. The reported values represent averages of five measurements. The particle size distributions of the neat Amitraz powder and the dispersions obtained after the completion of the dissolution experiments were determined.

### Scanning electron microscopy (SEM)

Amitraz powder was sputter coated with chromium and viewed using a Zeiss Supra 55 FEGSEM at 5 kV. The purpose was to determine the fundamental size of the individual particles.

### Consent for publication

All authors have given their consent for the submission of the manuscript.

## Data Availability

The authors are willing to share the data in Excel format with interested parties.
